# Self-reported frequency of sex as self-injury (SASI) in a national study of Swedish adolescents and association to sociodemographic factors, sexual behaviors, abuse and mental health

**DOI:** 10.1186/s13034-017-0146-7

**Published:** 2017-02-28

**Authors:** Cecilia Fredlund, Carl Göran Svedin, Gisela Priebe, Linda Jonsson, Marie Wadsby

**Affiliations:** 1 Child and Adolescent Psychiatry, Department of Clinical and Experimental Medicine, Faculty of Medicine, Linköping University, 581 85 Linköping, Sweden; 2 Barnafrid, Child and Adolescent Psychiatry, Department of Clinical and Experimental Medicine, Faculty of Medicine, Linköping University, 581 83 Linköping, Sweden; 3 Department of Psychology, Lund University, 221 00 Lund, Sweden

**Keywords:** Sex as self-injury (SASI), Non-suicidal self-injury (NSSI), Sexual abuse, Revictimization, Trauma, Self-harm, Indirect self-injury, Selling sex, Adolescents

## Abstract

**Background:**

Sex as self-injury has become a concept in Swedish society; however it is a largely unexplored area of research, not yet conceptualized and far from accepted in the research field. The use of sex as a way of affect regulation is known in the literature and has, in interviews with young women who sell sex, been compared to direct self-injury, such as cutting or burning the skin. The aim of this study was to investigate the self-reported frequency of sex as self-injury and the association to sociodemographic factors, sexual orientation, voluntary sexual experiences, sexual risk-taking behaviors, sexual, physical and mental abuse, trauma symptoms, healthcare for psychiatric disorders and non-suicidal self-injury.

**Methods:**

A representative national sample of 5750 students in the 3rd year of Swedish high school, with a mean age of 18 years was included in the study. The study was questionnaire-based and the response rate was 59.7%. Mostly descriptive statistics were used and a final logistic regression model was made.

**Results:**

Sex as self-injury was reported by 100 (3.2%) of the girls and 20 (.8%) of the boys. Few correlations to sociodemographic factors were noted, but the group was burdened with more experiences of sexual, physical and emotional abuse. Non-heterosexual orientation, trauma symptoms, non-suicidal self-injury and healthcare for suicide attempts, depression and eating disorders were common.

**Conclusions:**

Sex used as self-injury seems to be highly associated with earlier traumas such as sexual abuse and poor mental health. It is a behavior that needs to be conceptualized in order to provide proper help and support to a highly vulnerable group of adolescents.

## Background

Using sex as a means of self-injury has, during the last few years, been highlighted in Swedish media and by professionals working with adolescents [1, 2]. Sex as self-injury (SASI) has even been a term used in judgments in the Swedish Court of Appeal (Svea Hovrätt 2015: B2517). Few have described this behavior in research or in literature. In a report from the Children’s Welfare Foundation Sweden [1], sex as self-injury was suggested to be defined as: “when a person has a pattern of seeking sexual situations involving mental or physical harm to themselves. The behavior causes significant distress or impairment in school, work, or other important areas”. In the report, based on clinical experience and interviews with youths and professionals, a model for understanding repeated sexual risk-taking in the form of sex as self-injury, was presented. The core element behind SASI was in their model unbearable feelings, especially intense anxiety. An alternative definition for SASI was formulated by Stockholms Tjejjour, a Swedish non-profit organization working to help and support young females [2]. According to Stockholms Tjejjour, the definition of sex as self-injury is to have repetitive and recurrent intense feelings such as shame, guilt, anxiety, disgust and self-hatred that are confirmed and/or temporarily alleviated by repetitive and recurrent exposure to sexual and physical abuse, humiliation and violation. Alternatively, by the repetitive and recurrent search for sexual situations that distress and unease, that not necessarily, but often, involve a third party responsible for causing the physical and/or mental injury.

The above text and attempts at early definitions link the associations to a number of different areas such as self-injurious behavior in general, sexual risk-taking and the experience of traumatic events, especially sexual abuse.

### Self-injurious behaviors

Self-injurious behaviors (SIB) can either be direct, such as cutting or burning the skin, or indirect through the use of harmful behavior such as abusive relationships, binge eating or alcohol abuse [3, 4]. Direct self-injury is usually divided into suicidal and non-suicidal self-injury (NSSI) depending on the intention to kill oneself [5]. Earlier definitions of direct self-injurious behaviors have also included more indirect forms of self-injury such as risk-taking, promiscuity and drug abuse [3]. In a study based on 11 European countries the estimated lifetime prevalence of direct self-injurious behavior was 27.6%, occasionally seen in 19.7% and repetitively seen in 7.8%. The behavior was more common among girls [6]. In a review article from 2012, the mean prevalence for NSSI was estimated to 18.0% [7] and according to a Swedish study, 11.1% of girls and 2.3% of boys meet the DSM-5 criteria for NSSI syndrome [8].

Sexual risk-taking behaviors, substance abuse and eating disorders are usually considered to be an indirect form of SIB since they do not cause immediate damage to the body tissue and the effects may not be seen until later [9–11]. It has been suggested that to be considered as an indirect self-injurious behavior, the behavior should be repetitive, be of concern to clinicians or family members and potentially cause physical damage if continued [11]. Attention has recently been placed on shared factors for the co-occurrence of NSSI and indirect self-injury, such as eating disorders, with common elements seen in using the body to control state of mind and social situations [9, 12].

### Sexual risk-taking behaviors and affect regulation

During interviews, young women who sell sex have described using sex as a way to self-injure, in the same way as cutting or burning the skin [13]. Using sex as affect-regulation was described as follows by one woman who sold sex:

“When I feel bad I contact someone who wants to meet me. I feel so bad than that I’ll do just anything to relieve that pressure. Before the meetings the anxiety is so strong that I barley remember how I got there […] then I shut down. Let someone else take me over and decide. […]. Afterwards I feel like crap. Feel disgusting and empty. Often I am in a lot of pain. […].” [13, p. 23].

Sometimes self-injury through selling sex had even replaced cutting the skin as it was less visible. A further quotation from a young woman selling sex:

“[…] and I was the good one who didn’t self-harm anymore. Everyone was so pleased, but I felt just as bad, I just found other ways […] that weren’t that visible [selling sex] […] things that almost killed me for real.” [13, p. 23].

The self-destructiveness of selling sex and visiting online sex sites often increased in periods of poor mental health and the quitting process was described as challenging since the women found themselves caught in a behavior that was hard to break because of the function of affect regulation [13].

Associations between risky sexual behaviors and NSSI has been seen [13–15] and adolescents that have displayed risky sexual behaviors are twice as likely to have a history of suicide attempts [16]. Depressive symptoms independently predict risky sexual behavior in adolescents, indicating that sex is being used as a coping strategy for depression [17]. To use sexual intercourse as a way of affect regulation and coping strategy is a behavior that is known from the research field [17–21]. Using sex as a coping strategy was associated with younger women, more risky sexual behavior with poor condom use, experience of physical abuse during childhood or adolescence and poor communication with the partner [21].

### Child sexual abuse and sexual-risk behavior

Child sexual abuse is associated with later high-risk sexual behavior such as a greater number of sexual partners, higher frequency of sexually transmitted infections, teenage pregnancy, prostitution and earlier age of sexual debut [22–24]. Child sexual abuse also increases the risk of later sexual revictimization [19, 20, 22, 23, 25] which seems to be partly mediated by sexual self-esteem, sexual concerns and high risk sexual behavior [25]. The use of sexual intercourse as a way to reduce negative affects has been suggested as a pathway from sexual abuse during childhood or during adolescence, to later revictimization [19, 20]. Symptoms of depression and anxiety have been found to mediate the relationship between using sex as an affect-regulating strategy and sexual assault [18, 20]. The use of sex to reduce negative affects is associated with having more sexual partners, including more partners of casual nature [20]. Emotional dysregulation has been suggested as a direct pathway to revictimization, with risky sexual behavior as one resulting risk factor [26].

Since sex as self-injury (SASI) is a largely unexplored area of research, not yet conceptualized and far from accepted in the research field, there is a need to further explore its occurrence and associations to other behaviors and potential risk-factors.

## Aim of the study

The aim of this study was to investigate the self-evaluated prevalence of sex as self-injury (SASI) in a representative sample of adolescents in the 3rd year of the Swedish high school system. A second aim was to study the association between SASI and risk factors such as sociodemographic factors, sexual orientation, voluntary and risk-taking sexual behaviors, emotional, physical and sexual abuse and mental health through trauma symptoms, NSSI and the occurrence of seeking healthcare for psychiatric disorders.

In the present study, sex as self-injury is defined as a sexual behavior in relation to another person in order to self-injure.

## Methods

The study was a part of a national questionnaire-based survey called “Youths, Sex and Internet—in a changing world” and was performed at the request of the Swedish Ministry of Health and Social Affairs. The survey was partly a replication of two earlier studies that were carried out in 2004 and in 2009 [27, 28].

### Participants

The study was carried out in the 3rd and last year of Swedish high school during the fall of 2014. The selection of study sample, distribution and collection of the questionnaire was performed by Statistics Sweden (a national administrative agency). To form the study sample, the National School Register for the 2nd year of Swedish high schools for the fall of 2013 was used. By using stratification on the basis of school size and study program a total of 13,903 adolescents from 261 out of 1215 schools were selected for the study. Of the 261 schools selected, 238 were still providing the selected study programs in 2014. A total of 171 schools with 9773 adolescents agreed to participate in the study. Of the 9773 adolescents that had the opportunity to participate in the study, 5873 completed the questionnaire. Thirty-four questionnaires were excluded due to unserious answers or a high amount of missing data. This gave a response rate of 59.7%. A further 89 did not answer the index question about using sex as self-injury, resulting in a total of 5750 participants for the study. Mean age of the participating adolescents was 18.0 years (SD = .6). According to data from 2014, 91.7% of all Swedish 18 years old adolescents were enrolled in the Swedish high school system [29].

The study group was selected with the aim of being representative of the 3rd year of Swedish high schools. However, for a separate study concerning Stockholm, an extra sample from the county of Stockholm was included in the study. The additional Stockholm sample showed a lower response rate (48.7%) compared to the rest of the country (65.3%), came more often from middle-size schools with 191–360 pupils (51.2 vs. 41.6%), giving a small effect size (Cramer’s V = .10, χ^2^ = 63.6, *df* = 2, *p* = .000), and were more often studying practical high school programs (33.2 vs 27.7%), giving no effect size (Cramer’s V = .05, χ^2^ = 17.1, *df* = 1, *p* = .000). The Stockholm sample was retained in this study to give a larger and more robust study sample.

### Procedure

Information about the study was sent to the head of each school by mail. Students received written information about the study and gave informed consent for participation by filling in the questionnaire. According to the Ethical Review Act of Sweden, active consent is not required from parents of adolescents’ aged 15 years or older [30]. Participants answered the questionnaire in digital format (by computer, in 165 schools) or, where computers were not available, on paper (6 schools). Regardless of distribution method, anonymity was guaranteed. The study was performed during lecture time in the selected schools during September–November 2014. Reminders were given by phone during November 2014 to those schools that had not yet returned data. With regard to the sensitive topics in the questionnaire, both the head of the school, teachers responsible for the lecture and the participating adolescents received an information letter about the study including contact details for help and support if needed after answering the questionnaire.

### Measures

The questionnaire for the present study was a modified version of the questionnaire used in 2004 and 2009 [27, 28]. The questionnaire used for this study comprised in total 116 main questions, of which 34 were analyzed in the present study.

The *index question* for this study was new and literally formulated: “*Have you ever used sex to purposely hurt yourself?*”. To investigate the occurrence of sex as self-injury, questions included were; age at first occurrence, number of occurrences during the past year and in total, age and gender of the sexual encounter on the previous occasion and the perceived pain of using SASI.

Questions about *Sociodemographic factors* included gender with the options *boy*, *girl* and “*The classification ‘male’ or ‘female’ does not fit for me*”, parents’ occupation and education, financial situation in the family, immigrant background and living situation.

*Sexual behavior* and *sexual risk*-*taking*, were investigated by questions concerning sexual orientation, voluntary sexual experiences, age at first voluntary intercourse, number of sexual partners, use of contraceptives, occurrence of abortion (self or partner) and sexually transmitted infection of chlamydia. To investigate the occurrence of selling sex, the question used was “*Have you ever sold sexual services?*”.

The question related to *sexual abuse* was “*Have you been exposed to any of the following against your will?*”. Included in the options were: *someone having exposed him*-*/herself to you* via *the Internet or otherwise, someone having touched your genitals/tried to undress you to have sex with you, forced you to masturbate or have vaginal, oral or anal intercourse*. Flashing is by definition an abusive act according to Swedish law if it is against the will of the victim, irrespective of whether it occurs in real life or via the Internet, which is why it was included in the analysis for being exposed to ‘any sexual abuse’. Further analyses were made, including only penetrative abuse (oral/anal/vaginal abuse). Follow-up questions for sexual abuse were asked concerning the first exposure, as follows; age of the victim, relationship to the perpetrator and type of sexual abuse. One question was asked concerning the total number of times exposed to sexual abuse. All questions concerning sexual abuse were used in the questionnaires from 2004 and 2009.

Exposure to *emotional and physical abuse* was measured by the question; “*Have you prior to the age of 18 been subjected to any of the following by an adult?*”. Emotional abuse was measured through three questions; *insulted, threatened to be hit, isolated from friends*. Physical abuse was measured by eight questions, ranging from being pushed or shaken, hit with the hands or items, burned or strangled. The answers were ranked into four; *never*—*rarely*—*sometimes*—*often.* However, when analyzing the question the answers were dichotomized into ‘been exposed’ including the answers *rarely, sometimes* and *often*, or ‘never been exposed’. This instrument has not been validated but has been used in the earlier studies from 2004 and 2009.

*Contact with healthcare for psychiatric disorders* was measured with the question: “*Have you ever been in contact with healthcare services for…*” giving the following options: *Depression/anxiety, Eating disorders, ADHD/ADD or similar, Autism/Asperger, Suicide attempt, Alcohol/Drug abuse*. This question was new and formulated for this survey. The occurrence of NSSI was investigated with a general screening question: “*Have you ever done something to purposely hurt yourself without intending to die?”* This is a question included in the structural interview *Self*-*Injurious Thoughts and Behaviors Interview*—SITIB [31].

*Trauma symptoms* were measured by *Trauma Symptom Checklist for Children* (TSCC), an instrument designed to identify a broad range of trauma symptoms in children aged 8–17 years [32]. This is a widely-used self-report instrument for measuring trauma symptoms among children and adolescents [33] that has been used for adolescents up to 19 years of age [34–36]. The instrument comprises 54 items, divided into six subscales; anxiety, depression, post-traumatic stress (PTS), dissociation, anger and sexual concerns. Answers are arranged in the scale of four options *Never*—*Sometimes*—*Often*—*Almost all of the time.* Cronbach’s alpha coefficient for the subscales has been assessed to be .77 to .89 and .84 for the entire instrument [32]. There is a Swedish translation and validation for the 10–17 age group, giving a Cronbach’s alpha coefficient for the total scale of .94 with the variation of .78 to .83 for the subscales [37]. In the present study the Cronbach’s alpha was .95 for the total scale and .82 for anxiety, .88 for depression, .87 for PTS, .85 for dissociation, .84 for anger and .65 for sexual concerns.

### Analyses

Categorical data was presented using frequencies and cross tabulation and analyzed with Chi square test and Fisher’s Exact test using *p* value <.05. When comparing means such as age and TSCC, *t* test for independent groups was used. Percentages presented in the study relate to the number of adolescents answering the question. Missing answers in individual questions were at most 9.7%. Analyses by gender boy/girl were made but since the number of boys was very small, few statistically significant differences were found, indicating an increased risk of type II errors. Results are therefore presented divided by gender boy/girl only when statistical significance with a *p* value <.05 was seen. In the analyses the answer alternatives concerning living situation were merged from seven to four alternatives (living with both parents or alternating/living with one parent with or without new partner/alone or with siblings or partner/in foster care or institution), financial situation in the family from five to three alternatives (good/poor/do not know), sexual orientation from six to four alternatives (heterosexual/homosexual/bisexual/other or unsure), number of sexual partners from four to three alternatives (one, two to five and more than five). The questions concerning abortion and treatment for chlamydia were dichotomized from four to two alternatives (yes/no) and the question concerning total number of times exposed to sexual abuse was dichotomized from three to two alternatives (exposed one time/exposed more than one time). To make a model with the most important factors associated to SASI, forward stepwise binary logistic regression was performed with SASI as a dependent variable and sex, financial situation in the family, heterosexual sexual orientation, selling sex, all kinds of sexual abuse, penetrative abuse, emotional and physical abuse, trauma symptoms, healthcare for psychiatric disorders and NSSI, as covariates. All statistical analyses were carried out in Statistical Package of the Social Sciences (SPSS) version 22.

## Results

### Sex as self-injury

Of the total of 5750 students who answered the question about sex as self-injury, 125 (2.2%) stated that they had used SASI on at least one occasion, translating to 100 (3.2%) of the girls, 20 (.8%) of the boys and 5 (9.4%) of those who stated that the classification into male or female did not fit them. The mean age for first SASI was 15.6 (*SD* = 2.0) and the frequency of the behavior was reported with a median of 5 times. Within the previous 12 months, 58.5% had used SASI 1–5 times, 16.3% more than 5 times and 25.2% had not used SASI during the previous year. The sexual encounter was for girls in 96.9% of cases with a male and in 90.9% of the cases with someone in the age 15–25 years. The sexual encounter was for boys in 52.9% of the cases with a female and in 60.0% with someone in the age 15–25 years. Pain during the SASI was perceived by 70.7% of the girls. For 39.4%, this was sharp or moderate pain. Pain was perceived by 55.6% of the boys, of whom 27.8% experienced moderate pain.

### Sociodemographic data

Sociodemographic data for adolescents using SASI and reference adolescents not using SASI (non-SASI) are presented in Table 1. The group of adolescents using SASI had a generally poorer family financial situation, fewer lived with both parents and more often alone, with siblings or partner, in foster care or institution. No differences were seen concerning parents being in employment, parents’ education, immigrant background, attending theoretical or practical study program or being enrolled at a school in the county of Stockholm.

**Table 1:** Sociodemographic factors for adolescents using sex as self-injury (SASI) and adolescents not using sex as self-injury (non-SASI)

	SASI n = 123–125		Non-SASI n = 5599–5625		Total n = 5724–5750		χ^2^	*df*	*p value*
	*n*	*%*	*n*	*%*	*n*	*%*			
Total nr of participants	125	2.2	5625	97.8	5750	100			
Gender							50.9	2	<.001
Boy	20	16.0	2522	44.8	2542	44.2			
Girl	100	80.0	3054	54.3	3154	54.9			
“This division doesn’t fit me”	5	4.0	48	.9	53	.9			
Study program									ns
Theoretical	84	67.2	4002	71.1	4086	71.1			
Practical	41	32.8	1623	28.9	1664	28.9			
Adolescents from Stockholm	35	28.0	1531	27.2	1566	27.2			ns
Fathers working	106	86.2	4931	87.9	5037	87.9			ns
Mothers working	103	83.1	4894	87.3	4997	87.2			ns
Fathers with university education	44	35.2	2262	40.4	2306	40.3			ns
Mothers with university education	59	47.2	2932	52.3	2991	52.1			ns
Family financial situation							26.6	2	<.001
Good	83	66.4	4477	79.6	4560	79.3			
Poor	42	33.6	952	16.9	994	17.3			
Do not know	0	.0	193	3.4	193	3.4			
Adolescents with immigrant background	11	8.8	489	8.7	500	8.7			ns
Mothers with immigrant background	25	20.0	1219	21.7	1244	21.6			ns
Fathers with immigrant background	26	20.8	1221	21.7	1247	21.7			ns
Living situation							53.3	3	<.001
With both parents or alternating	61	48.8	4036	71.8	4097	71.3			
With one parent with or without new partner	37	29.6	1194	21.3	1231	21.4			
Alone or with siblings or partner	23	18.4	358	6.4	381	6.6			
In foster care or institution	4	3.2	32	.6	36	.6			

### Sexual orientation

Adolescents using SASI, more frequently reported sexual-minority orientation, as seen in Table 2. Only 60% of the adolescents in the SASI group had a heterosexual orientation compared to 88% in the reference group, 5.6% of the index group reported homosexual orientation, 23.8% bisexual orientation and 11.2% had another sexual orientation or were unsure of their sexual orientation.

**Table 2:** Sexual orientation, voluntary sexual experiences and sexual risk-taking behavior in adolescents using sex as self-injury (SASI) or not (non-SASI)

	SASI n = 119–125		Non-SASI n = 3654–5625		Total n = 3773–5750		χ^2^	*df*	*p value*
	*n*	*%*	*n*	*%*	*n*	*%*			
Sexual orientation							124.5	3	<.001
Heterosexual	75	60.0	4943	87.9	5018	87.3			
Homosexual	7	5.6	74	1.3	81	1.4			
Bisexual	29	23.2	240	4.3	269	4.7			
Other or unsure	14	11.2	368	6.5	382	6.6			
Voluntary sexual experiences									
Oral intercourse	115	92.0	3232	58.0	3347	58.7	58.4	1	<.001
Anal intercourse	65	52.4	1025	18.5	1090	19.2	89.8	1	<.001
Vaginal intercourse	112	89.6	3441	61.8	3553	62.4	40.3	1	<.001
Total number of sexual partners^a^							66.2	2	<.001
One	12	10.1	1186	32.5	1198	31.8			
2–5	40	33.6	1572	43.0	1612	42.7			
More than 5	67	56.3	896	24.5	963	25.5			
Use of contraceptives last intercourse^a^	70	58.3	2500	68.3	2570	68.0	5.3	1	.021
Ever experiences abortion (self or partner)	10	8.0	216	3.8	226	3.9			.031*
Ever treatment for Chlamydia	7	5.6	214	3.8	221	3.8			ns
Ever sold sexual services	14	11.3	37	.7	51	.9			<.001*

* Fisher’s exact test

^a^Questions about number of sexual partners and contraceptive use have only been asked to adolescents with earlier voluntary sexual experiences

### Voluntary sexual experiences

As seen in Table 2, adolescents using SASI displayed more experiences of voluntary sexual intercourse, had more sexual partners and an earlier sexual debut, at an age of 14.6 (*SD* = 1.7) years compared to 15.6 (*SD* = 1.5) years among the non-SASI group (*t* = −6.11, *p* < .001).

### Sexual risk-taking behavior

Adolescents using SASI were slightly less likely to have used contraceptives at last intercourse and slightly more likely to have had an abortion, but no difference was seen for being treated for chlamydia. Selling sex was seen among 11.3% of the adolescents using SASI compared to 0.7% among non-SASI, see Table 2.

### Child abuse

As seen in Table 3, 75.0% of the adolescents using SASI had been exposed to some kind of sexual abuse. When divided by gender this was 35.0% in the SASI group for boys compared to 9.2% in the non-SASI group and 82.8% among girls in the SASI group compared to 27.5% in the non-SASI group. Differences were especially prominent concerning penetrative abuse. Of the adolescents that had been exposed to sexual abuse, 73.1% of the SASI group had been exposed more than once compared to 54.3% in the non-SASI group (χ^2^ = 12.2, *df* = 1, *p* < .001). The first occurrence of sexual abuse was at the younger age of 13.8 (*SD* = 2.8) years for the SASI group compared to 14.6 (*SD* = 2.7) years in the non-SASI group (*t* = −2.8, *p* = .005) and was more commonly penetrative sexual abuse [47.3% (*SASI*) vs 19.7% (*non*-*SASI*), χ^2^ = 36.8, *df* = 1, *p* < .001]. The perpetrator on the first occasion was more commonly a boyfriend/girlfriend or former boyfriend/girlfriend for adolescents using SASI [25.8% (*SASI*) vs 11.8% (*non*-*SASI*), χ^2^ = 14.6, *df* = 1, *p* < .001], and not a stranger [22.6% (*SASI*) vs 38.8% (*non*-*SASI*), χ^2^ = 9.5, *df* = 1, *p* = .003]. For 52 (58.4%) of the 89 adolescents reporting their age at the time of sexual abuse and SASI, sexual abuse preceded the use of sex as self-injury. This figure was 81 (91%) when including adolescents reporting the same year for the first experience of sexual abuse and SASI.

**Table 3:** Sexual abuse, emotional and physical abuse among adolescents using sex as self-injury (SASI) or not (non-SASI)

	SASI n = 122–125		Non-SASI n = 5180–5611		Total n = 5304–5736		χ^2^	*df*	*p * value
	*n*	*%*	*n*	*%*	*n*	*%*			
Sexual abuse									
*Any sexual abuse*	93	75.0	1014	19.6	1107	20.9	225.3	1	<.001
Only penetrative	59	48.4	290	5.3	349	6.2	379.7	1	<.001
Emotional abuse									
*Any emotional abuse*	109	87.2	3206	57.1	3315	57.8	45.3	1	<.001
Insult	106	84.8	3028	54.0	3134	54.7	46.9	1	<.001
Threats of hitting	55	44.0	1081	19.3	1136	19.8	47.1	1	<.001
Isolation from friends	48	38.4	908	16.2	956	16.7	43.4	1	<.001
Physical abuse									
*Any physical abuse*	86	69.4	1697	30.3	1783	31.2	86.2	1	<.001
Pushed, shaken	70	56.0	1293	23.1	1363	23.8	73.2	1	<.001
Throw something	40	32.3	738	13.2	778	13.6	37.7	1	<.001
Hit with hands	52	41.6	782	13.9	834	14.5	75.2	1	<.001
Kick, bite, hit with fist	21	16.8	310	5.5	331	5.8	28.6	1	<.001
Hit with objects	13	10.5	191	3.4	204	3.6			<.001*
Burn, scald	7	5.6	100	1.8	107	1.9			.009*
Strangle	22	17.6	197	3.5	219	3.8			<.001*
Other physical assault	42	34.1	440	7.9	482	8.4	107.8	1	<.001

* Fisher’s exact test

Adolescents using SASI were more often exposed to some form of emotional or physical abuse, as seen in Table 3. Within the SASI group, 87.2% had been exposed to some form of emotional abuse compared to 57.1% in the non-SASI group. Exposure to some form of physical abuse was seen among 69.4% in the SASI group compared to 30.3% among peers in the non-SASI group.

### Trauma symptoms, non-suicidal self-injury and psychiatric disorders

As seen in Table 4, trauma symptoms measured by the subscales for anxiety, depression, post-traumatic stress, dissociation, anger and sexual concerns in TSCC were all more common in the adolescents using SASI. NSSI was seen among 65.6% in the SASI group compared to 16.6% in the group of non-SASI, see Table 5. Contact with healthcare services for depression/anxiety, eating disorders, ADHD/ADD or similar, autism/Asperger, suicide attempt, alcohol and drug abuse was sought to a much higher extent in the SASI group compared to non-SASI, as seen in Table 5. Of the adolescents using SASI, 61% had sought help for depression/anxiety, 31.4% for suicide attempt and 28.8% for eating disorders.

**Table 4:** Trauma symptom measured though Trauma Symptom Checklist for Children (TSCC) for adolescents using sex as self-injury (SASI) or not (non-SASI)

	SASI n = 123–124		Non-SASI n = 5514–5515		Total n = 5635–5639		
	Mean	SD	Mean	SD	Mean	SD	*p * value
Anxiety	9.6	5.5	4.6	3.9	4.7	4.0	<.001
Depression	12.4	6.2	5.0	4.4	5.2	4.6	<.001
Anger	9.0	5.7	4.0	4.0	4.2	4.1	<.001
Post-traumatic stress	14.0	6.4	6.1	4.9	6.2	5.1	<.001
Dissociation	12.9	6.2	5.9	4.8	6.0	4.9	<.001
Sexual concerns	4.8	3.9	2.2	2.4	2.6	2.5	<.001

**Table 5:** Non-suicidal self-injury (NSSI) and Healthcare for psychiatric disorders among adolescents using sex as self-injury (SASI) or not (non-SASI)

	SASI n = 117–125		Non-SASI n = 5442–5618		Total n = 5559–5743		χ^2^	*df*	*p * value
	*n*	*%*	*n*	*%*	*n*	*%*			
Non-suicidal self-injury	82	65.6	933	16.6	1015	17.7	201.7	1	<.001
Healthcare for psychiatric disorders									
Depression/anxiety	75	61.0	1005	18.2	1080	19.2	141.8	1	<.001
Eating disorders	34	28.8	289	5.3	323	5.8		1	<.001
ADHD/ADD or similar	21	17.6	387	7.1	408	7.3	116.8	1	<.001
Autism/Asperger	8	6.8	100	1.8	108	1.9			.002*
Suicide attempt	38	31.4	191	3.5	229	4.1	19.2		<.001*
Alcohol/drug abuse	14	12.0	111	2.0	125	2.2			<.001*

* Fishers exact test

### Binary logistic regression analyses

A forward stepwise binary logistic regression with SASI as the dependent variable was performed to find a model with the most important variables associated with the behavior. The model included in nine steps. In the final model, the most important factors associated with the behavior were selling sex, some kind of sexual abuse, penetrative sexual abuse, physical abuse, TSCC for dissociation, NSSI, healthcare for depression/anxiety and eating disorders. The value for TSCC and depression was not significant (*p* = .060) but was left in the statistic model. The variables of sex, financial situation in the family, heterosexual orientation, emotional abuse, trauma symptoms for anxiety, anger, PTS, sexual concerns and healthcare for psychiatric disorders for ADHD/ADD or similar, Autism/Asperger, suicide attempt and alcohol/drug abuse were all significantly associated with SASI in pairwise chi-2 statistics, but were not left in the final model of the binary logistic regression (Table 6).

**Table 6:** Binary logistic regression, final outcome for forward stepwise analyses made in 9 steps with sex as self-injury (SASI) as dependent variable

	B	S.E.	Wald	p value	aOR	95% CI
Selling sex	1.61	.47	12.0	.001	5.00	2.01–12.42
Any sexual abuse	1.12	.29	14.6	<.001	3.06	1.72–5.44
Penetrative sexual abuse	1.26	.27	21.4	<.001	3.52	2.06–6.00
Physical abuse	.68	.24	7.9	.005	1.97	1.23–3.17
TSCC depression	.06	.03	3.5	.060	1.06	1.00–1.12
TSCC dissociation	.07	.03	7.1	.008	1.08	1.02–1.13
Healthcare for depression/anxiety	.64	.25	6.7	.010	1.90	1.17–3.10
Healthcare for eating disorders	.76	.28	7.4	.006	2.15	1.24–3.71
Non suicidal self-injury	.66	.25	6.8	.009	1.94	1.18–3.19

Cox & Snell R^2^ .071

Nagelkerke R^2^ .370

## Discussion

To our knowledge, this study is the first that has attempted to investigate the prevalence of sex as self-injury (SASI) and its association to sociodemographic factors, sexual behaviors, experiences of abuse and mental health. The results of this study can be summarized in five main findings.

*First*, sex as self-injury was used, according to their own definition, by 3.2% of the girls and .8% of the boys—within the 3rd year of Swedish high school. The findings indicate that sex is used as a way of self-injury although we do not know the exact definition of SASI in the view of the adolescents answering the question since the adolescents did not have to state the kind of sexual activity concerned when using SASI. What is clear is that all the sexual activities were in a sexual encounter and 70% of the girls and 55% of the boys experienced pain on the most recent occasion. The adolescents using SASI did not have a higher risk of sexually transmitted infection of chlamydia, only a slightly higher risk of abortion and slightly lower use of contraceptives but other forms of risk-taking sexual behavior were seen such as more voluntary sexual behaviors, more sexual partners and higher frequency of selling sex, as reported by 11.3%. Selling sex has been described as a way of self-injury through having the same function of reducing anxiety as cutting the skin and even replacing the cutting of the skin since it is less visible [13]. To gain a better understanding of how and why sex is used as self-injury a qualitative study is planned to investigate the manifestations and motives of SASI.

*Second*, there was a clear association between SASI and other types of direct and indirect self-injurious behaviors such as NSSI, drug abuse, eating disorders and suicide attempts. Seeking healthcare for suicide attempts was as common as 31.4% among the adolescents using SASI. Prior studies found adolescents with sexual risk-taking behaviors being twice as likely to have attempted suicide [16]. This is also in line with interviews with young women selling sex, who describe themselves as living very close to death and fearing for their lives as a result of committing suicide or being killed in a sex-selling meeting [13]. It is also supported by co-occurrence of self-injurious behaviors such as NSSI and eating disorders [9] and the correlation of risky sexual behaviors and NSSI [15, 38]. The high incidence of self-injurious behaviors in the group of adolescents with SASI indicates a group of adolescents using different strategies to cope with affect regulation. Together with TSCC scores indicating much more trauma symptoms and the finding that 61% had sought healthcare for depression or anxiety disorders we see that this is a vulnerable group that needs to be highlighted in the healthcare system, so that they can receive proper help and support.

*Third*, social and demographic data seem to be weakly correlated with SASI. The same finding has been made for adolescents selling sex where social and demographic correlations have been few in Sweden and Norway. Differences have been seen in adolescents selling sex and living situations/parental divorce as seen in the present study of adolescents using SASI. Findings for selling sex and financial situation in the family, immigrant background and parental education have been inconsistent in different studies [39–41]. However, to have bisexual or homosexual orientation was associated to SASI, for both boys and girls, that is a group that needs to be highlighted. This finding is in line with studies of adolescents selling sex and adolescents with NSSI [41, 42].

*Fourth,* the adolescents using SASI had more often been exposed to penetrative sexual abuse and they were more often revictimized. They had had more voluntary sexual experiences, earlier age of first intercourse, more sexual partners and reported to have sold sexual services to a higher extent. As mentioned in the introduction, child sexual abuse is associated with later high-risk sexual behavior such as a greater number of sexual partners, higher frequency of sexually transmitted infections, teenage pregnancy, prostitution and earlier age of sexual debut [22–24]. Emotional dysregulation predict high risk sexual behaviors and has been seen as a mediator for revictimization after exposure for child sexual abuse [26]. Sex used as a way to reduce negative affects has been suggested as a pathway from child sexual abuse or sexual abuse during adolescence to later revictimization [19, 20]. From prior studies is the connection between sexual abuse and high-risk sexual behavior known, including prostitution, and the risk of using sex as a way of emotional dysregulation leading to a higher risk of revictimization. The question is, could the risk of revictimization after sexual abuse be partly explained by emotional dysregulation when using SASI?

*Fifth*, as seen in the logistic regression, the most strongly associated variables with the behavior were selling sex, sexual abuse, physical abuse, dissociation, NSSI and seeking healthcare for eating disorders and depression. These results could be interpreted as explanatory variables such as childhood abusive experiences while dissociation, selling sex, eating disorders and NSSI could be seen as co-existing variables to SASI. Sexual abuse and NSSI have not always been associated [43] but this finding has been inconsistent [44]. What we see in this study is a close connection between SASI and sexual abuse. Of the girls, 82.8% had been exposed to some form of sexual abuse. Of the adolescents that had been exposed to sexual abuse, 91% had been exposed before, or within the same year that they started to use SASI. Could the sexual abuse be the leading cause for using sex as a way of self-injury rather than using other kinds of NSSI, such as cutting or burning the skin? Child sexual abuse could lead to the feeling of the body being “damaged goods” [45] and in interviews with young women selling sex it has been described that the body could be used as a tool and was of no value, explaining the view that it could be ‘hurt’ and used for selling sex [13].

### Limitations

The results of this study are intended to be representative of adolescents in the 3rd year of Swedish high school. It was decided to perform the study during lecture times rather than send questionnaires by mail, to improve response rate and consequently the level of representation of Swedish adolescents. However, only 59.7% of the eligible adolescents answered the questionnaire which could be compared to the previous studies from 2004 and 2009 that had a response rate of 77.2 and 60.4% respectively. Of the missing answers, approximately 10% are explained by those being absent on a regular school day. The rest of the missing answers are those that chose not to participate which might be due to poor motivation, a feeling of discomfort when answering the questions in the questionnaire, insufficient knowledge of the Swedish language or not having the ability to focus for the time needed to answer the questionnaire. Conclusively, the adolescents that did not answer the questionnaire might have been a more exposed group, thereby making our findings not representative and more likely to be underestimated than exaggerated.

The strongest limitation of the study is a lack of definition of SASI and that we do not know in which way the participants have purposely hurt themselves by using sex. The definition for using SASI is in this study self-defined and more studies are needed to confirm or reject the concept and the reported number of using it.

## Conclusion

To summarize, 2.2% of Swedish adolescents in the 3rd year of high school report that they have used SASI at some point and this was more common among girls. The group of adolescents using SASI report a higher incidence of different kinds of self-injurious behaviors such as NSSI, drug abuse and suicide attempts. Correlations to sociodemographic factors were few but SASI was strongly associated with sexual abuse. That sex is being used as self-injury could, at least partly, be explained by the feeling of the body being damaged goods following exposure to sexual abuse, thereby leading to the use of the body as a tool in sexual encounters in addition to other self-injurious behaviors. This, however, needs to be elaborated on in further studies, including those using qualitative methods. Trauma symptoms, depression or anxiety disorders indicate a group in need of help and support, which is why it is important to conceptualize the behavior so it can be addressed in the healthcare system.

## Abbreviations

ADD: attention deficit disorder
ADHD: attention deficit hyperactivity disorder
DSH: deliberate self-harm
DSM: diagnostic and statistical manual of mental disorders
Non-SASI: adolescents not using sex as self-injury
NSSI: non suicidal self-injury
PTS: post-traumatic stress
SASI: sex as self-injury
SD: standard deviation
SE: standard error
SIB: self-injurious behaviors
SITIB: self-injurious thoughts and behaviors interview
SPSS: Statistical Package of the Social Sciences
TSCC: trauma symptom checklist for children
